# Inflammasome Proteins in Serum and Serum-Derived Extracellular Vesicles as Biomarkers of Stroke

**DOI:** 10.3389/fnmol.2018.00309

**Published:** 2018-09-04

**Authors:** Nadine Kerr, Marta García-Contreras, Sam Abbassi, Nancy H. Mejias, Brandon R. Desousa, Camillo Ricordi, W. Dalton Dietrich, Robert W. Keane, Juan Pablo de Rivero Vaccari

**Affiliations:** ^1^Department of Neurological Surgery, The Miami Project to Cure Paralysis, University of Miami, Miami, FL, United States; ^2^Diabetes Research Institute, Miller School of Medicine, University of Miami, Miami, FL, United States; ^3^Department of Physiology and Biophysics, University of Miami, Miami, FL, United States; ^4^InflamaCORE, LLC, Miami, FL, United States

**Keywords:** inflammasome, biomarkers, extracellular vesicles, serum, exosomes, caspase-1, ASC

## Abstract

The inflammasome is a key contributor to the inflammatory innate immune response after stroke. We have previously shown that inflammasome proteins are released in extracellular vesicles (EV) after brain and spinal cord injury. In addition, we have shown that inflammasome proteins offer great promise as biomarkers of central nervous system (CNS) injury following brain trauma. In the present study, we used a Simple Plex Assay (Protein Simple), a novel multi-analyte automated microfluidic immunoassay platform, to analyze serum and serum-derived EV samples from stroke patients and control subjects for inflammasome protein levels of caspase-1, apoptosis-associated speck-like protein containing a caspase-recruitment domain (ASC), Interleukins (IL)-1β, and (IL)-18. Receiver operator characteristic (ROC) curves with associated confidence intervals obtained from the analysis of serum samples revealed that the area under the curve (AUC) for ASC was 0.99 with a confidence interval between 0.9914 and 1.004, whereas the AUC for caspase-1, IL-1β, and IL-18 were 0.75, 0.61, and 0.67, respectively. Thus, these data indicate that ASC is a potential biomarker of stroke and highlight the role of the inflammasome in the inflammatory response after brain ischemia.

## Introduction

The innate immune response is a well-known contributor to the inflammatory response after stroke (Xu and Jiang, 2014; Brand et al., 2015; Neumann et al., 2015). We have previously shown that the inflammasome, an arm of the innate immune response involved in the activation of caspase-1 and the pro-inflammatory cytokines interleukin (IL)-1β and IL-18, contributes to the pathology of stroke (Abulafia et al., 2009). Moreover, therapeutic targeting of the inflammasome has been shown to improve outcomes after Central Nervous System (CNS) injury, including stroke (Brand et al., 2016). In addition, extracellular vesicles (EV) such as exosomes contain inflammasome proteins that contribute to the spread of inflammation after brain trauma and spinal cord injury (de Rivero Vaccari et al., 2016a) as well as stroke (Ji et al., 2016; Zhang and Chopp, 2016).

A biomarker is a characteristic that can be measured objectively and evaluated as an indicator of normal or pathologic biological processes (Biomarkers Definitions Working Group, 2001). Thus, biomarkers in blood or other body fluids can be used as indicators of stroke onset. However, to date, there is no biomarker available that is used in the diagnosis and management of stroke. Cytokines such as IL-10 or tumor necrosis factor as well as other inflammatory proteins like C-reactive protein (CRP), high-mobility group box-1 or heat shock proteins have been considered as potential biomarker candidates in stroke patients (Whiteley et al., 2009; Katan and Elkind, 2011; Bustamante et al., 2016).

Inflammasome proteins have a great potential to be used as biomarkers of injury and disease (Adamczak et al., 2012). In this study we analyzed serum and serum-derived EV samples from stroke patients and from control subjects, and calculated receiver operator characteristic (ROC) curves and associated confidence intervals to establish the suitability of inflammasome signaling proteins as biomarkers of stroke. In addition, we evaluated different EV isolation methods in order to determine the most suitable method to isolate EVs containing inflammasome proteins.

## Materials and methods

### Participants

In the present study, we analyzed serum samples from 80 age-matched control subjects and 16 patients that were diagnosed with stroke and donated their blood in the chronic phase after their stroke onset. Samples were purchased from Bioreclamation*IVT*. Subjects were enrolled in the study “Prospective Collection of Samples for Research” according to IRB # 201209495 approved by Schulman Associates IRB for Bioreclamation*IVT*. The control subject group consisted of samples obtained from 40 male and 40 female donors in the age range of 46–70 years old. The age range in the stroke group consisted of samples obtained from patients in the age range of 46–87 years old (Table 1).

**Table 1:** Characteristics of subjects with stroke included in the study.

**Age Range**	**Diagnosis**	**Medications**
60–70	Stroke, Hypertension (HTN), Hyperlipidemia	Avodart; Diovan; Flomax; Fosamax; Lantus; Plavix; Toprol; Vitoryn; Cilostazol; Ciprofloxacin; Diltia; Foradil; hydrochlorothiazide (HCTZ); Micardis
60–70	Stroke, Age Related Macular Degeneration (AMD), Hypothyroidism, Osteogenesis Imperfecta, Hypertension (HTN)	Plavix; Synthroid; Norvasc; Lovastatin; Hydroxyurea; Flomax
50–60	Stroke, Seizures	Dilantin; Ibuprofen; Botox injections
60–70	Stroke, Caverous Angioma, Allergy (Codine), Hypertension (HTN), Hypercholesterolemia	Amlodipine; ASA; Enalapril; Labetalol; Simvastatin
80–90	Gout, Chronic Kidney Disease (CKD), Osteoarthritis, Chronic Obstructive Pulmonary Disease (COPD), Insomnia, Hypertension (HTN), Benign Prostatic Hyperplasia (BPH), Gastritis, Coronary Artery Disease (CAD), Peripheral Artery Disease, Anemia, Dyslipidemia, Arteriosclerotic Disese, Renal Cyst, Sigmoid Diverticulosis, Gastritis, Ischemic Stroke, Hypertensive Heart Disease	Diovan 160 mg, Diltiazem 300 mg, Crestor 20 mg, Plavix 75 mg, Flomax 0.4 mg, Fluticasone 50 mg, Hydrochlorothiazide 12.5 mg, Ranitidine 150 mg, Pantoprazole 40 mg, Metoprolol 50 mg, Finasteride 5 mg, Temazepam 15 mg, Carbidopa 15 mg, Allopurinol 100 mg
60–70	Ovarian Cancer, Hypertension (HTN), Stroke	Phenergan 25 mg, Compazine 10 mg, Oxycodone 5 mg
70–80	Ovarian Cancer, Hydronephrosis, Leukocystosis, Normocytic Anemia, Stroke	Lipitor 40 mg, Colace 100 mg, Ferrous Sulfate 325 mg, Buspar 5 mg, Plavix 75 mg, Folic Acid 1 mg, Emla, Lisinopril 10 mg, Oxycodone-Acetaminophen 5–325 mg, Vitamin B6, Vitamin B12 1,000 mcg
40–50	Donor with Fever, Hypercholesterolemia, Cardiovascular Disease, Stroke	Advil, Lipitor, Plavix
80–90	Congestive Heart Failure (CHF), Prostate and Skin Cancer, Mini stroke patient, Osteoarthritis, Conn's Syndrome	None
80–90	Type 1 Diabetes, Age Related Macular Degeneration (AMD), Neuropathy, Lumbar Disc Herniation, Stroke	Pletal, Humalog, Lantus, alprazolam, Fluoxetine, crestor, metroprolol, lisinipril, glipizide, praxacid, lyrica, metanx, magnesium sulfate, omega 3, tylenol, asprin, cilostavol, antioxidant
80–90	Stroke, Myocardial Infarction (MI), Branch Retinal Vein Occlusion	Levothyroxine, Indapamide, Plavix, Cozar, Metformin
70–80	Donor on Heparin, Hypertension (HTN), End Stage Renal Disease (ESRD), Stroke	Aranesp 40 mcg, Zemplar 2 mcg, Venofer 10 mg, Amlodipine 10 mg, Colace 100 mg, Lasix 40 mg, Metoprolol 100 mg, Heparin 1000iu
50–60	Donor on Heparin, Hyperlipidemia, Asthma, Coronary Artery Disease (CAD), Hypertension (HTN), End Stage Renal Disease (ESRD), Stroke	Aranesp 2.5 mcg, Zemplar 7 mcg, Renvela 3,200 mg, Simvastatin 20 mg, Benazepril 5 mg, Albuterol 25 mg, Folic Acid 1 mg, Budesonide 0.5 mg, Pantoprazole 40 mg, Heparin 3500iu
60–70	Gout, Hypertension (HTN), Stroke, Dyslipidemia, Peripheral Vascular Disease, Coronary Artery Disease (CAD), Chronic Obstructive Pulmonary Disease (COPD), Type 2 Diabetes	Allopurinol 100 mg, Alprazolam 1 mg, Atorvastatin 20 mg, Lisinopril 2.5 mg, Metoprolol 50 mg, Ferrous Sulfate 325 mg
70–80	Hypertension (HTN), End Stage Renal Disease (ESRD), Stroke	Renvela 800 mcg, Calcitriol 0.5 mg
50–60	Non-Small Cell Lung Cancer (NSCLC), Dyslipidemia, Hypertension (HTN), Stroke	Acetaminophen-Codeine, Avastin, Folic Acid, Prochlorperazine, Promethazine, Amlodipine 2.5–10 mg, Aspirin 81 mg, Lipitor 40 mg, Plavix 75 mg, Stress Formula, Tylenol w/Codeine

### Isolation of EV from serum

EV were extracted from serum by two different commercially available methods.

- **Total Exosome Isolation reagent (from Serum) kit (Invitrogen):** Total Exosome Isolation reagent (from serum) was used according to the manufacturer's instructions (Invitrogen). Briefly, 100 μl of each sample was centrifuged at 2,000 × g for 30 min. The supernatant was then incubated with 20 μl of Total Exosome Isolation reagent for 30 min at 4°C followed by centrifugation at 10,000 × g for 10 min at room temperature. Supernatants were discarded and the pellet was resuspended in 50 μl of PBS.
- **ExoQuick precipitation:** EV were isolated from serum samples using ExoQuick (EQ, System Biosciences) as previously described in de Rivero Vaccari et al. (2016a). Briefly, 100 μl of each sample was centrifuged at 3,000 × g for 15 min. The supernatant was then incubated with 24.23 μl of ExoQuick Exosome Precipitation Solution (for serum) for 30 min at 4° C followed by centrifugation at 1,500 × g for 30 min. Supernatants were discarded and residual EQ solution was centrifuged at 1,500 × g for 5 min. The pellet was then resuspended in 50 μl of PBS.

### Simple plex assay

To determine the protein concentration of caspase-1, ASC, IL-1β, and IL-18 in serum and serum-derived EV, a Simple Plex assay was run using the Ella System (Protein System) as described (Brand et al., 2016). Accordingly, samples were diluted 1:1 and 50 μl of each diluted sample was loaded into a well of an Ella CART. Two milliliters of washing buffer were also loaded in the appropriate well, and the assay was analyzed by the Simple Plex Explorer software. Results correspond to the mean of each sample run in triplicates.

### Protein quantification

To quantify the protein concentration in isolated EV, the Pierce Coomassie (Bradford) Protein Assay Kit (ThermoFisher Scientific, Inc.) was used according to manufacturer's instructions. Accordingly, 5 μl of each standard or unknown sample was loaded into a 96 well-plate while 250 μl of Coomassie Reagent was added to each well. The plate was then incubated for 10 min at room temperature and the absorbance measured at 595 nm with a SPARLK 10M (TEKAN) spectrophotometer. Serum-derived EV were then lysed (1:1 dilution) in lysis buffer as previously described (de Rivero Vaccari et al., 2016a).

### Nanoparticle tracking analysis (NTA)

The concentration and size distribution of the isolated exosomes was analyzed by NanoSight NS300 system (Malvern Instruments Company, Nanosight, and Malvern, United Kingdom). Briefly, EV preparations were homogenized by vortexing followed by serial dilution of 1:1,000 in sterile Phosphate buffer saline (PBS) and analyzed with the NanoSight NS300. Each sample analysis was conducted for 90 min. Data were analyzed by Nanosight NTA 2.3 Analytical Software (Malvern Instruments Company, Nanosight, and Malvern, United Kingdom) with the detection threshold optimized for each sample and screen gain at 10 to track as many particles as possible with minimal background. Polystyrene latex standards were analyzed to validate the operation of the instrumentation and a blank 0.2 μm-filtered 1x PBS was also run as a negative control. At least three analysis were done for each individual sample.

### Immunoblotting

For detection of inflammasome signaling proteins in isolated EV, EV were resuspended in protein lysis buffer and resolved by immunoblotting as previously described (de Rivero Vaccari et al., 2015). Briefly, following lysis of the pellet, proteins were resolved in 10–20% Criterion TGX Stain-Free precasted gels (Bio-Rad), using antibodies (1:1,000 dilution) to NLRP3 (Novus Biologicals, cat# IMG-6763A), caspase-1 (Novus Biologicals, cat# NB100-56565), ASC (Santa Cruz, cat# sc-271054), IL-1β (Cell Signaling, cat# 12242S), IL-18 (Abcam, cat# ab71495), CD81 (Life Technologies, cat# MA5-13548) and NCAM (Sigma, cat# C9672). Quantification of band density was done using the UN-SCAN-IT gel 5.3 Software (Silk Scientific Corporation). Ten microliters of sample was loaded. Chemilluminescence substrate (LumiGlo, Cell Signaling) in membranes was imaged using the ChemiDoc Touch Imaging System (BioRad).

### Gel imaging

Total protein in the Criterion TGX Stain-Free precasted gels was imaged using the ChemiDoc Touch Imaging System (BioRad) by placing the gel in the tray of the ChemiDoc Touch following protein transfer. The image was then adjusted in the screen to show the entirety of the gel and running the Stain-Free Blot setting in the application window.

### Statistical analyses

Statistical comparisons between the Invitrogen and ExoQuick isolation procedures were done using a two-tailed student *t*-test.

### Biomarker analyses

Data were analyzed using Prism 7 software (GraphPad). Comparisons between groups for protein levels were carried by first identifying outliers followed by an unpaired *t*-test and then determining the area under the ROC curve, as well as the 95% confidence interval and the *p*-value (*p*-value of significance used was <0.05). Finally, sensitivity, specificity, positive predictive value (PPV), negative predictive value (NPV) and accuracy of each biomarker was obtained for a range of different cut-off points. Samples that yielded a protein value below the level of detection of the assay were not included in the analyses for that particular analyte.

## Results

### Caspase-1, ASC, and IL-18 are elevated in the serum of stroke patients

**Figure 1:**
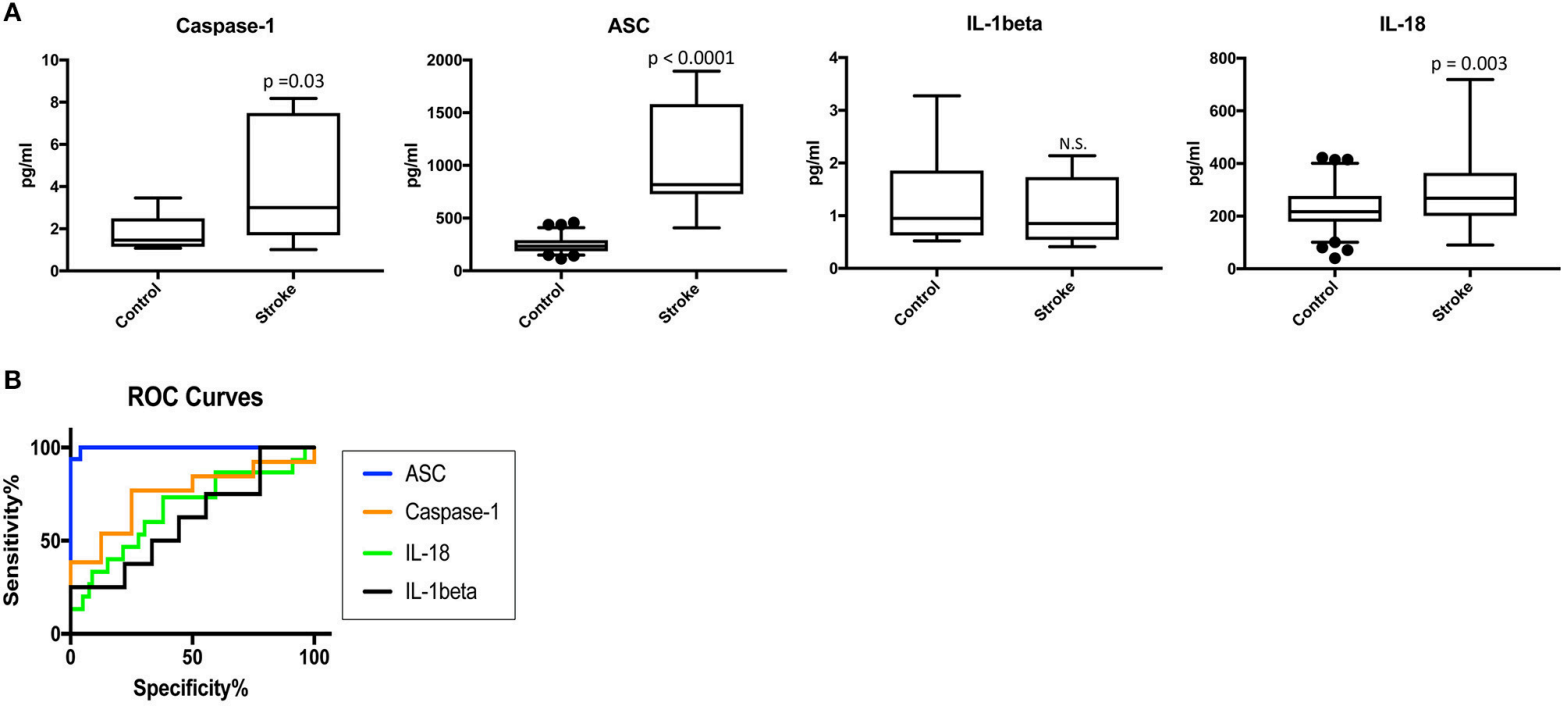
Inflammasome proteins are elevated in the serum of stroke patients. **(A)** Protein levels in pg/ml of caspase-1 ASC, IL-1β and IL-18 analyze by with a Simple Plex system in serum samples from patients with stroke and healthy donors. *p*-value of significance is shown above each box plot. Box and whiskers are shown for the 5th and 95th percentile. N.S., Not Significant. Caspase-1: *N* = 8 control and *N* = 13 stroke; ASC: *N* = 75 control and *N* = 16 stroke; IL-1: *N* = 9 control and *N* = 8 stroke; and IL-18: *N* = 79 control and *N* = 15 stroke. **(B)** ROC curves for caspase-1 (orange), ASC (blue), IL-1β (black) and IL-18 (green). Caspase-1: *N* = 8 control and *N* = 13 stroke; ASC: *N* = 75 control and *N* = 16 stroke; IL-1: *N* = 9 control and *N* = 8 stroke; and IL-18: *N* = 79 control and *N* = 15 stroke. Dark circles correspond to data points outside the 95% confidence interval.

To determine the protein levels of inflammasome proteins in serum from stroke patients and control subjects, we analyzed serum samples with a Simple Plex system. Protein levels of caspase-1, ASC, IL-1beta and IL-18 were analyzed. Protein levels of caspase-1, ASC and IL-18 were higher in the serum of stroke patients when compared to the control samples, whereas levels of IL-1β were not significantly different (Figure 1A). Thus, these findings potentially show a positive correlation between caspase-1, ASC and IL-18 with stroke.

### ASC as a serum biomarker of stroke

Higher levels of inflammasome proteins in serum from stroke patients is not enough proof to show that inflammasome proteins are potential biomarkers of stroke. Thus, we performed an ROC analysis (Figure 1B and Supplementary Figure 1) to determine the AUC. ROC analysis was carried using GraphPad Prism 7 software with the ROC Curve Built-in analysis mode with the settings of 95% Confidence Interval and Report results as percentage, which provides the area, standard error, confidence interval and *p*-value, as well as sensitivity and specificity for a variety of cut-off points and respective likelihood ratios and ROC curve. The AUC for caspase-1, IL-18 and IL-1beta was 0.75 or below. However, the AUC for ASC was 0.9975 with a confidence interval between 0.9914 to 1.004 (Table 2). The cut-off point for ASC was 404.8 pg/ml with a sensitivity of 100% and a specificity of 96% (Table 2). Thus, ASC appears to be a reliable biomarker of stroke.

Table 2ROC analysis results for inflammasome signaling proteins in serum and cut-off point analyses for inflammasome signaling proteins in serum.

**Table:** 

**Biomarker**	**Area**	**Std. Error**	**95% C.I**.	***P*-value**
Caspase-1	0.75	0.1087	0.5369 to 0.9631	0.05
ASC	0.9975	0.003	0.9914 to 1.004	<0.0001
IL-1beta	0.6111	0.1407	0.3353 to 0.8869	0.44
IL-18	0.6675	0.082	0.5059 to 0.8291	0.04

**Table:** 

**Biomarker**	**Cut-off point (pg/ml)**	**Sensitivity (%)**	**Specificity (%)**
Caspase-1	> 1.412	85	50
ASC	> 404.8	100	96
IL-1beta	< 0.984	63	56
IL-18	> 244.6	73	62

### Amount of protein loaded in isolated EV from stroke patients

To calculate the amount of protein present in the isolated EV from serum samples, we performed a protein assay from isolates obtained by the Invitrogen method and the ExoQuick method. Our data indicate that with the ExoQuick method we were able to isolate more overall protein than with the Invitrogen method (Supplementary Figure 2A). To visualize how much protein was loaded in a gel during immunoblot analysis, we used the Stain-Free Blot setting of the ChemiDoc Touch Imaging System. The representative image in Supplementary Figure 2B shows that when we loaded 10 μl of the serum-derived EV resuspended in lysis buffer containing a protease inhibitor cocktail (Sigma), the lanes corresponding to the Invitrogen kit had less protein than the lane corresponding to the ExoQuick kit; however, there was no statistical significant difference between the groups (Supplementary Figure 2C).

### Invitrogen's and exoquick kits isolate CD81- and NCAM-positive EV from the serum of patients with stroke

**Figure 2:**
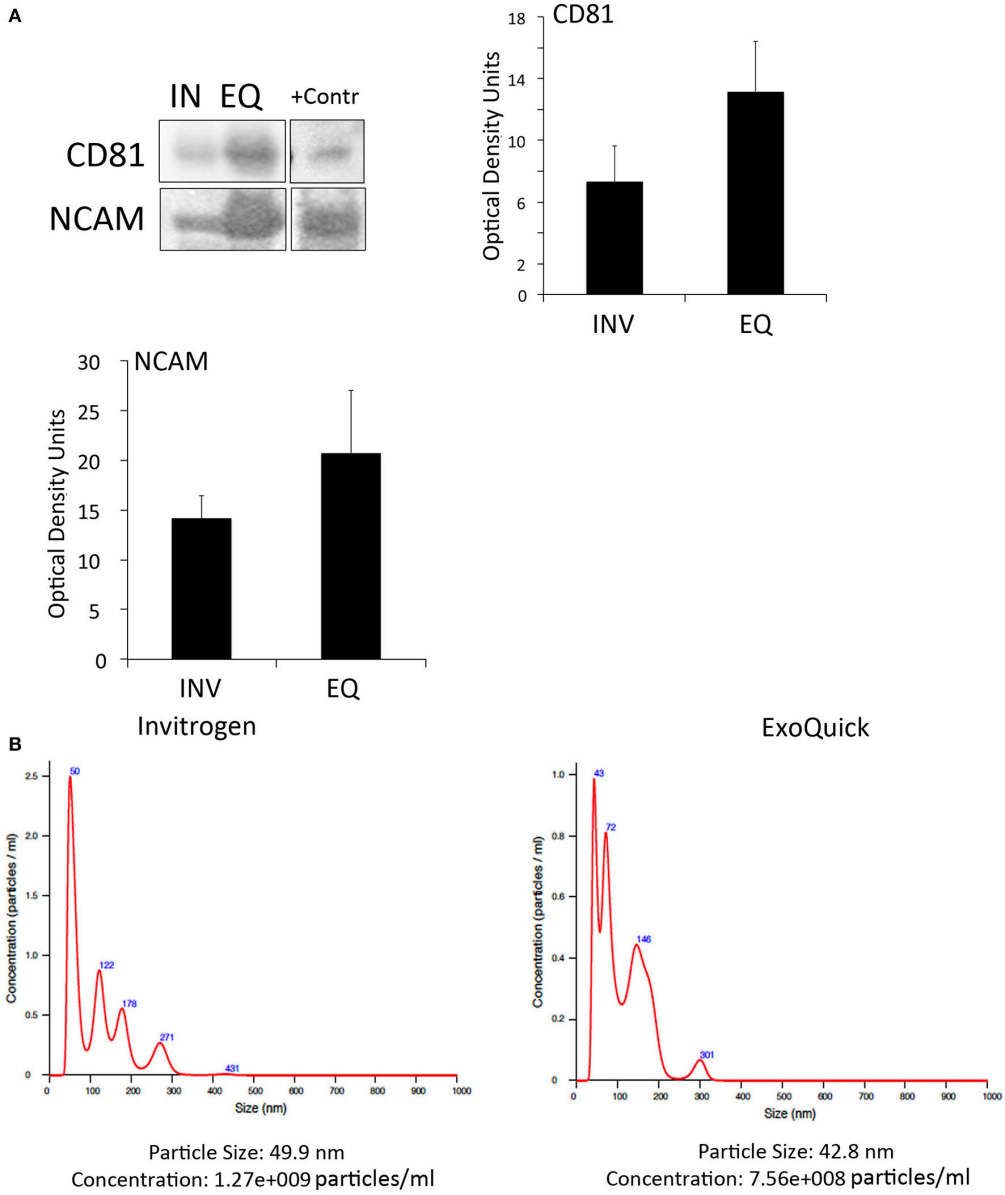
EV characterization in serum from stroke patients**. (A)** Representative immunoblot of CD81 and NCAM positive EV isolated with the Invitrogen Kit (IN) and the ExoQuick Kit (EQ). +Contr: Positive control of isolated EV. Quantification of CD81- and NCAM-positive EV isolated from serum with the Invitrogen kit (INV) and the ExoQuick kit (EQ). Nanoparticle tracking analysis of isolated serum-derived EV. **(B)** Nanoparticle tracking analysis predicts size distribution and concentration of particles in serum-derived EV samples isolated with the Invitrogen kit and the ExoQuick kit.

To determine if inflammasome proteins present in EV are promising biomarkers of stroke, we isolated EV from the serum of stroke patients. We used two different techniques of EV isolation to identify the most suitable method to isolate, inflammasome-containing EV. In addition we characterized by immunuoblot the expression of the tetraspanin protein CD81, a marker of EV (Andreu and Yáñez-Mó, 2014), as well as the expression of neural cell adhesion molecule (NCAM), a marker of neuronal-derived EV, to demonstrate that the isolated EV are indeed brain derived (Vella et al., 2016). Accordingly, both methods, the one from Invitrogen and ExoQuick, were able to isolate CD81- and NCAM-positive EV (Figure 2A). However, although the ExoQuick method seems to isolate higher levels of these proteins, there was no statistical significant difference between the two groups (Figure 2A). An EV-positive control isolate (System Biosciences) was run in parallel.

In addition, NTA analyses (Supplementary Videos 1, 2) revealed that the particle size was in the 40 to 50 nm range for both techniques, and the particle concentration of EV with the Invitrogen method was 1.27e+009 particles/ml and with ExoQuick, 7.56+008 particles/ml (Figure 2B). Thus, it seems that in our hands, both methods are suitable to isolate EV.

### Invitrogen's kit and exoquick isolate inflammasome-positive EV from the serum of patients with stroke

We have previously shown that inflammasome proteins are present in EV (de Rivero Vaccari et al., 2016a). We then compared the protein levels of caspase-1, ASC, IL-1β, NLPR3, and IL-18 by the two different methods and found no statistical significant difference in NLPR3, caspase-1, ASC and IL-18 levels between the two different methods. However, the ExoQuick method was able to isolate EV with higher levels of IL-1β than the Invitrogen method (Supplementary Figure 3).

### ASC is elevated in EV isolated from the serum of stroke patients

**Figure 3:**
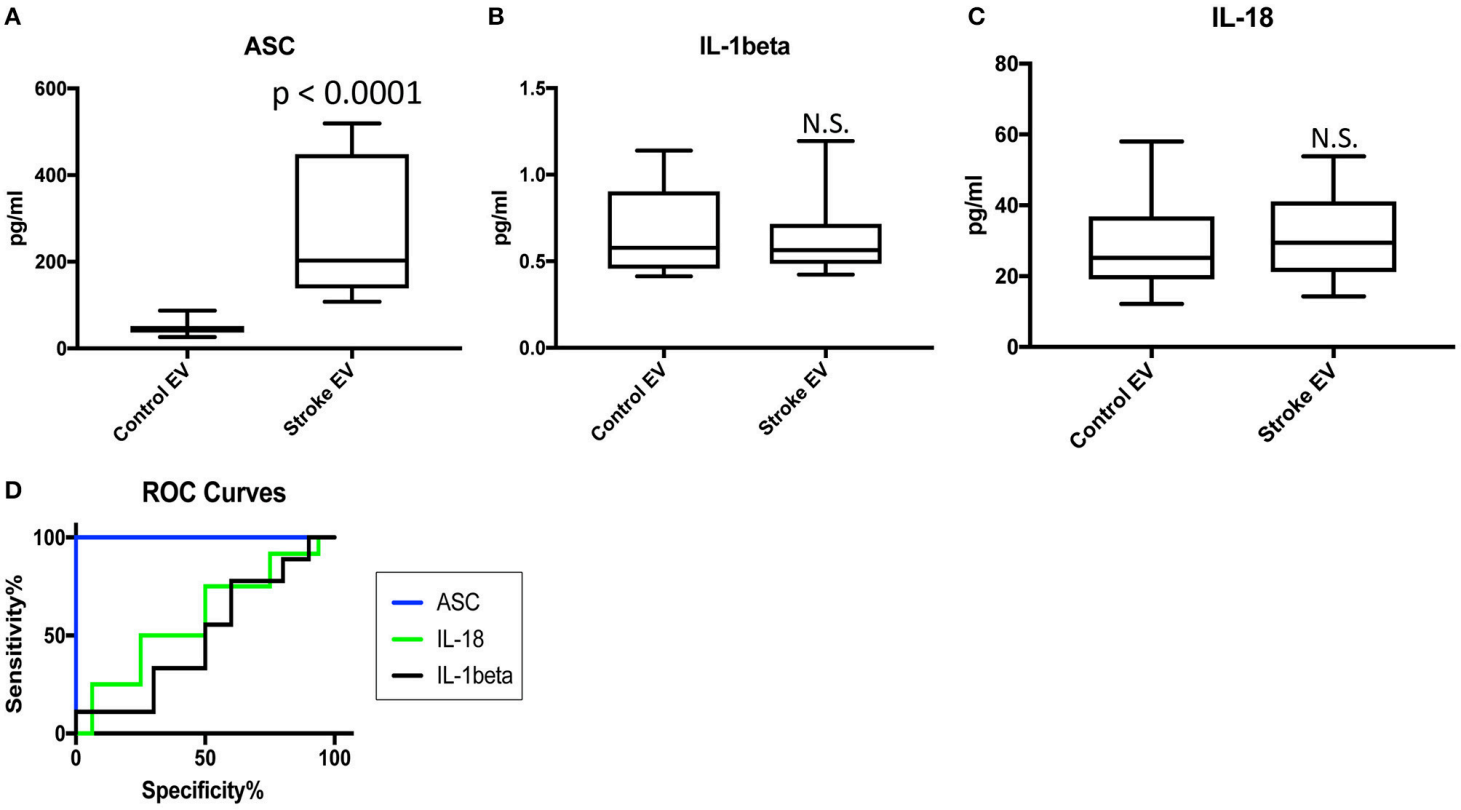
ASC is elevated in serum-derived EV of stroke patients. Protein levels in pg/ml of ASC **(A)**, IL-1β **(B)** and IL-18 **(C)** in serum-derived EV from patients with stroke and healthy donors. *p*-value of significance is shown above each box plot. Box and whiskers are shown for the 5th and 95th percentile. N.S., Not Significant. ASC: *N* = 16 control and 16 stroke; IL-1β: *N* = 10 control and 9 stroke; and IL-18: *N* = 16 control and 13 stroke. **(D)** ROC curves for ASC (blue), IL-1β (black) and IL-18 (green). ASC: *N* = 16 control and 16 stroke; IL-1β: *N* = 10 control and 9 stroke; and IL-18: *N* = 16 control and 13 stroke.

We then isolated EV from the serum of 16 aged-matched donors and 16 stroke samples (Table 1) and analyzed the protein levels of caspase-1, ASC, IL-1β, and IL-18 in these isolated EV with the Simple Plex technology. The protein levels of ASC remained higher in serum-derived EV from stroke samples when compared to controls (Figure 3). However, the levels of IL-1β and IL-18 were not significantly different between the two groups, while the levels of caspase-1 in these isolated EV was below the limit of detection of this assay for this analyte.

### ASC in serum-derived EV is a good biomarker of stroke

To determine if inflammasome proteins in serum-derived EV can be viable biomarkers of stroke, we then carried an ROC analysis (Supplementary Figure 4), and found that ASC is a reliable biomarker of stroke (Figure 3) with an AUC of 1 and a cut-off point of 97.57 pg/ml (Table 3).

Table 3ROC analysis results for inflammasome signaling proteins in serum-derived EV and cut-off point analyses for inflammasome signaling proteins in serum-derived EV.

**Table:** 

**Biomarker**	**Area**	**Std. Error**	**95% C.I**.	***P*-value**
ASC	1	0	1	< 0.0001
IL-1beta	0.5	0.1375	0.2303 to 0.7697	>0.9999
IL-18	0.5938	0.1109	0.3763 to 0.8112	0.4034

**Table:** 

**Biomarker**	**Cut-off point (pg/ml)**	**Sensitivity (%)**	**Specificity (%)**
ASC	>97.57	100	100
IL-1beta	>0.5585	56	50
IL-18	>23.66	75	50

## Discussion

Previous work on the inflammasome has indicated that inflammasome proteins can be used as biomarkers after traumatic brain injury (Adamczak et al., 2012) and multiple sclerosis (Keane et al., 2018). The inflammasome is a multiprotein complex of the innate immune response involved in the activation of caspase-1 and the processing of the inflammatory cytokines IL-1β and IL18. The inflammasome contributes to the inflammatory response after injury to the brain and the spinal cord, among others (de Rivero Vaccari et al., 2008, 2009, 2014, 2015). Thus, in this study we aimed at gaining a better understanding regarding the potential of inflammasome signaling proteins as biomarkers of stroke incidence in serum and serum-derived EV. Moreover, in this study we also analyzed different methods of EV isolation in order to develop a suitable and reliable method to isolate EV containing inflammasome proteins.

Inflammasome activation plays a critical role in the pathogenesis of stroke. For instance, the NLRP1 and the NLRP3 inflammasome have been shown to be activated by thromboembolic stroke (Abulafia et al., 2009; Fann et al., 2013). The NLRP2 inflammasome, which was initially described in human astrocytes (Minkiewicz et al., 2013), also contributes to the inflammatory response after cerebral ischemia in mice (Sun et al., 2016). Following intracerebral hemorrhage, the NLRP3 inflammasome plays a role in the infiltration of neutrophils (Ma et al., 2014). In addition, other pathways have been suggested to modulate the inflammatory response. For example, estrogen receptor-β signaling modulates inflammasome activation after global cerebral ischemia (de Rivero Vaccari et al., 2016b), and MAPK and NFκ-B signaling also appears to promote inflammasome activation (Fann et al., 2017). Thus, these findings highlight the importance of the inflammasome in the pathogenesis of stroke and the potential implications of the inflammasome as a therapeutic target after cerebral ischemia (Abulafia et al., 2009; Zhang et al., 2014).

There are 17.5 million deaths related to cardiovascular disease every year, of which 6.7 million occur as a result of stroke (Mendis et al., 2015). Atherosclerosis is a main contributor to stroke onset, and the formation of atherosclerotic plaques are in part the results of a deregulated inflammatory response that affects endothelial cells (Esenwa and Elkind, 2016). Atherosclerosis is an inflammatory/lipid-based disease, and the NLRP3 inflammasome has been considered as a link between lipid metabolism and inflammation because crystalline cholesterol and oxidized low-density lipoprotein (oxLDL), two abundant components in atherosclerotic plaques, activate the NLRP3 inflammasome (Hoseini et al., 2018). In addition, lipoprotein-associated phospholipase A2 (Lp-PLA2) also accumulates in atherosclerotic lesions and is released in response to inflammation (Rosenson and Stafforini, 2012). Thus, inflammatory proteins are good candidate biomarkers of stroke (Ridker and Haughie, 1998). The Rotterdam study demonstrated a correlation between Lp-PLA2 and the risk of stroke (Oei et al., 2005). Furthermore, Brain Natriuretic Peptide (BNP) has been associated with cardioembolic stroke. D-dimer, a degradation product from fibrin, also has a predictive value as a biomarker of stroke. Accordingly, elevated levels of D-dimer have been associated with stroke progression (Barber et al., 2004). Similarly, fibrinogen also has the potential to be used as a biomarker of stroke outcome (Turaj et al., 2006). Even though there have been some large studies of stroke biomarkers, there is yet to be a gold standard biomarker that is used in the care of stroke patients.

In this study, we used ROC curves as the AUC. An AUC of 1.0, signified that 100% of subjects in the population are correctly classified as having a stroke. However, an AUC of 0.5 means that subjects can randomly be classified as either positive or negative for stroke, thus, an AUC of 0.5 has no clinical utility. Importantly, an AUC between 0.9 and 1.0 demonstrates that such molecule is an excellent biomarker; from 0.8 to 0.9, good; 0.7 to 0.8 fair; 0.6 to 0.7, poor and 0.5 to 0.6, fails (Xia et al., 2013). Here our study shows the area under the curve (AUC) for ASC in serum was 0.9975 with a confidence interval between 0.9914 to 1.004. This AUC value was higher than caspase-1 (0.75), IL-1β (0.6111), and IL-18 (0.6675), indicating that ASC is a superior biomarker to the other proteins. The cut-off point for ASC was 404.8 pg/ml with 100% sensitivity and 96% specificity with the cohort of samples used. Importantly, we were able to increase the AUC to 1 when analyzing serum-derived EV samples from a small subset of patients. Accordingly, the cut-off point for ASC in serum-derived EV was found to be 97.57 pg/ml.

Future studies will be designed to evaluate the levels of inflammasome proteins as biomarkers of stroke outcome, as well as biomarkers of stroke risk and of respond to therapeutic intervention, and data will be compared to other potential stroke biomarkers. Importantly, the protein levels of caspase-1 in serum-derived EV were below the level of detection of the assay used in the present study. Therefore, future studies should be completed using a more sensitive assay method to determine the levels of caspase-1 in serum and EV.

Standards for Reporting Diagnostic accuracy studies (STARD) guidelines were used in this study. Based on these standards, limitations of this study include that samples correspond to different time points (4 months or even longer) pertaining to the chronic phase after stroke. Thus, although these results indicate that the proteins analyzed in this study remain elevated chronically after stroke, future studies are needed to address how long these proteins remain elevated within the chronic phase by careful analysis of samples taken at later collection points. In addition, it will be important to measure the levels of these proteins acutely after stroke, as well as the long-term effects that an acute elevation in inflammasome proteins cause after stroke. Lastly, another limitation that should be considered is the presence of co-morbidities in the patient population used in this study, for these can also contribute to increased inflammasome protein expression, yet the common denominator in all the samples used in this study is that all patients had stroke.

The chronic presence of inflammasome proteins after stroke suggests that there is a long-term inflammatory response chronically after stroke. This inflammation may also contribute to long-term damage as a result of stroke. In addition, from a diagnostic point of view, our findings suggest that potentially ASC and caspase-1 may be used to diagnose stroke even beyond the acute phase.

Inflammasome proteins are released in EV isolated from cerebrospinal fluid of brain and spinal cord injured patients (de Rivero Vaccari et al., 2016a). Inflammasome protein levels were higher in EV isolated from brain-injured patients when compared to control samples (de Rivero Vaccari et al., 2016a), thus indicating that they may be used as novel biomarkers in the diagnosis and prognosis of CNS injuries. EV may be derived from a variety of cells such as dendritic cells (dexosomes), neurons (synaptic vesicles), bone or cartilage (Le Pecq, 2005; Mathivanan et al., 2010). These particles range in diameter from 20 nm to 5 μm, and are composed of proteins, lipids and nucleic acids such as microRNAs (Kourembanas, 2015). EV have been identified in serum, plasma, urine, cerebrospinal fluid, saliva, breast milk and ascites (Théry et al., 2002; de Rivero Vaccari et al., 2016a) and play a role in innate and adaptive immune signaling. EV bind to surface molecules in recipient cells and then by endocytosis or membrane fusion, the contents of the exosome are passed on to the recipient cell (Théry et al., 2009). Thus, EV carry molecules from one cell to another and play a key role in cell signaling. EV have been implicated in neurodegenerative diseases (Vella et al., 2008), infections (Izquierdo-Useros et al., 2010), cancer (Luga et al., 2012), and in immune signaling (Robbins and Morelli, 2014). This study shows that EV released in serum after stroke carry an important cargo of inflammasome proteins that may play a role in innate immune inflammatory responses observed in stroke patients.

In this study, we used two methods to isolate EV from the serum of stroke patients: The Total Exosome Isolation from Serum kit (Invitrogen) and the ExoQuick (SBI System Biosciences). ExoQuick (EQ) Precipitation Solutions have been shown to be as effective as ultracentrifugation (UC) in isolating miRNA (Rekker et al., 2014). Importantly, the System Biosciences method is less time consuming than UC. Moreover, the EQ Precipitation method has been shown to be a more suitable method for EV isolation in regards to reproducibility and efficiency when compared to UC (Caradec et al., 2014). In the present study, both methods yielded EV containing inflammasome proteins and EV of consistent size as determined by NTA analysis. Moreover, EV were NCAM positive, suggesting that EV released after stroke are of neural origin and may be released after brain damage caused by stroke.

In summary, our findings highlight the potential use of inflammasome proteins, particularly ASC, as biomarkers of stroke in serum and serum-derived EV. Future studies, will determine if inflammasome proteins have a prognostic value of stroke severity and outcome.

## Author contributions

All authors were involved in the design of experiments. NK, MG-C, SA, NM, BDS, and JdRV carried the experiments that contributed to the data in this manuscript. All authors were involved in the analysis of the data, and all authors contributed to writing this manuscript and approved the manuscript prior to submission.

### Conflict of interest statement

JdRV, RK, and WD are co-founders and managing members of InflamaCORE, LLC and have patents on inflammasome proteins as biomarkers of injury and disease as well as on targeting inflammasome proteins for therapeutic purposes.

The remaining authors declare that the research was conducted in the absence of any commercial or financial relationships that could be construed as a potential conflict of interest.

## Supplementary material

Supplementary Figure 1 ROC curves for caspase-1 **(A)**, ASC **(B)**, IL-1β **(C)**, and IL-18 **(D)** from serum samples of stroke and healthy donors.

Supplementary Figure 2 **(A)** Comparison of total protein levels from serum-derived EV. A Bradford Assay was carried following EV isolation from serum to determine total protein concentration in isolates with the Invitrogen kit (INVTR) and the ExoQuick kit (EQ). Data presented as mean ± SEM. *N* = 6 per group. **(B)** Representative image of total protein loaded. Stain-free image of serum-derived EV proteins. Equal amounts of protein lysates (10 μl) were loaded in each lane of a Criterion gel. **(C)** Bar graph shows quantification of the entire lane corresponding to loaded EV isolated with the Invitrogen kit (INV) and the ExoQuick kit (EQ).

Supplementary Figure 3 Characterization of inflammasome proteins in serum-derived EV. Representative image of immunoblot analyses of inflammasome proteins in EV from serum. Quantification of immunoblot analysis of NLRP3, caspase-1, ASC, IL-1β, and IL-18 in EV derived from serum using the Invitrogen kit (IN) and the ExoQuick kit (EQ). Data presented as mean ± SEM. *N* = 6 per group. ^*^*p* < 0.05.

Supplementary Figure 4 ROC curves for ASC **(A)**, IL-1β **(B)**, and IL-18 **(C)** from serum samples of stroke and samples corresponding to the control subject group.

Supplementary Table 1 Raw data used for the analysis of this paper.

Supplementary Video 1 Representative video of EV isolated with the Invitrogen method.

Supplementary Video 2 Representative video of EV isolated with the ExoQuick method.
