# Supplementary figures and images for: Inflammasome Proteins in Serum and Serum-Derived Extracellular Vesicles as Biomarkers of Stroke
Source: Front Mol Neurosci. 2018 Sep 4;11:309. doi: 10.3389/fnmol.2018.00309 (PMC6131639; doi:10.3389/fnmol.2018.00309)

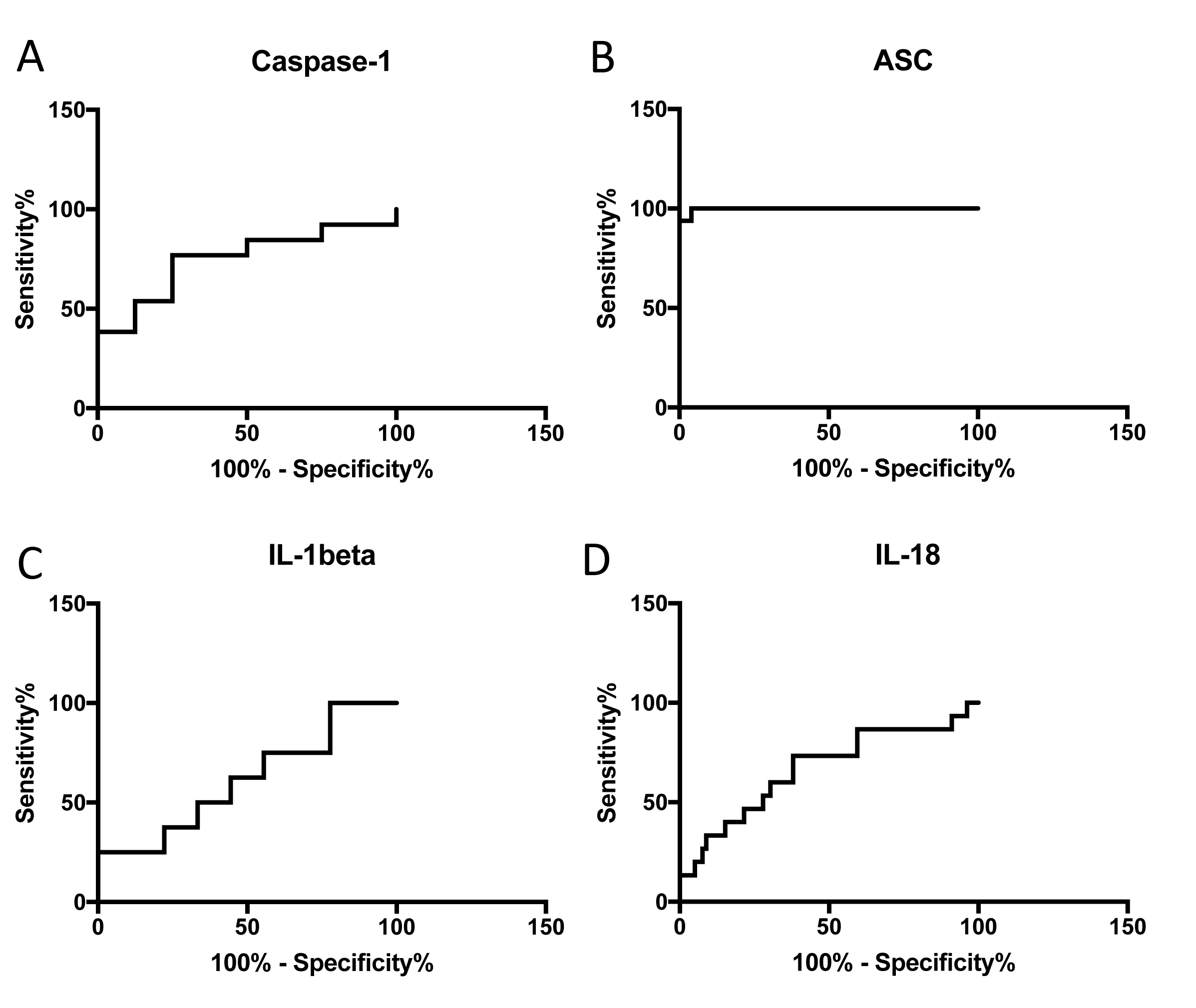

Supplement: Supplementary Figure 1 — ROC curves for caspase-1 (A), ASC (B), IL-1β (C), and IL-18 (D) from serum samples of stroke and healthy donors. [file Image_1.JPEG]

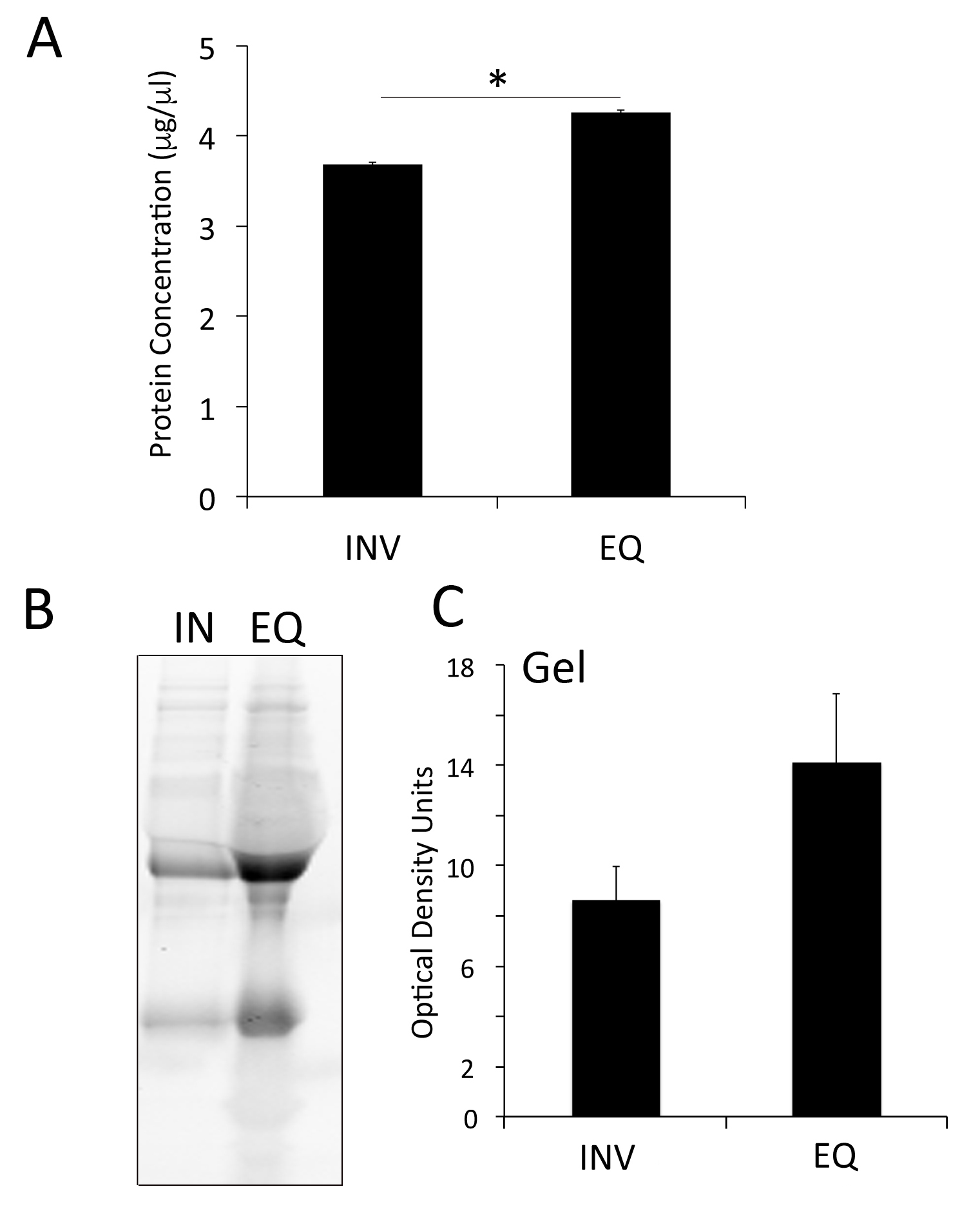

Supplement: Supplementary Figure 2 — (A) Comparison of total protein levels from serum-derived EV. A Bradford Assay was carried following EV isolation from serum to determine total protein concentration in isolates with the Invitrogen kit (INVTR) and the ExoQuick kit (EQ). Data presented as mean ± SEM. N = 6 per group. (B) Representative image of total protein loaded. Stain-free image of serum-derived EV proteins. Equal amounts of protein lysates (10 μl) were loaded in each lane of a Criterion gel. (C) Bar graph shows quantification of the entire lane corresponding to loaded EV isolated with the Invitrogen kit (INV) and the ExoQuick kit (EQ). [file Image_2.JPEG]

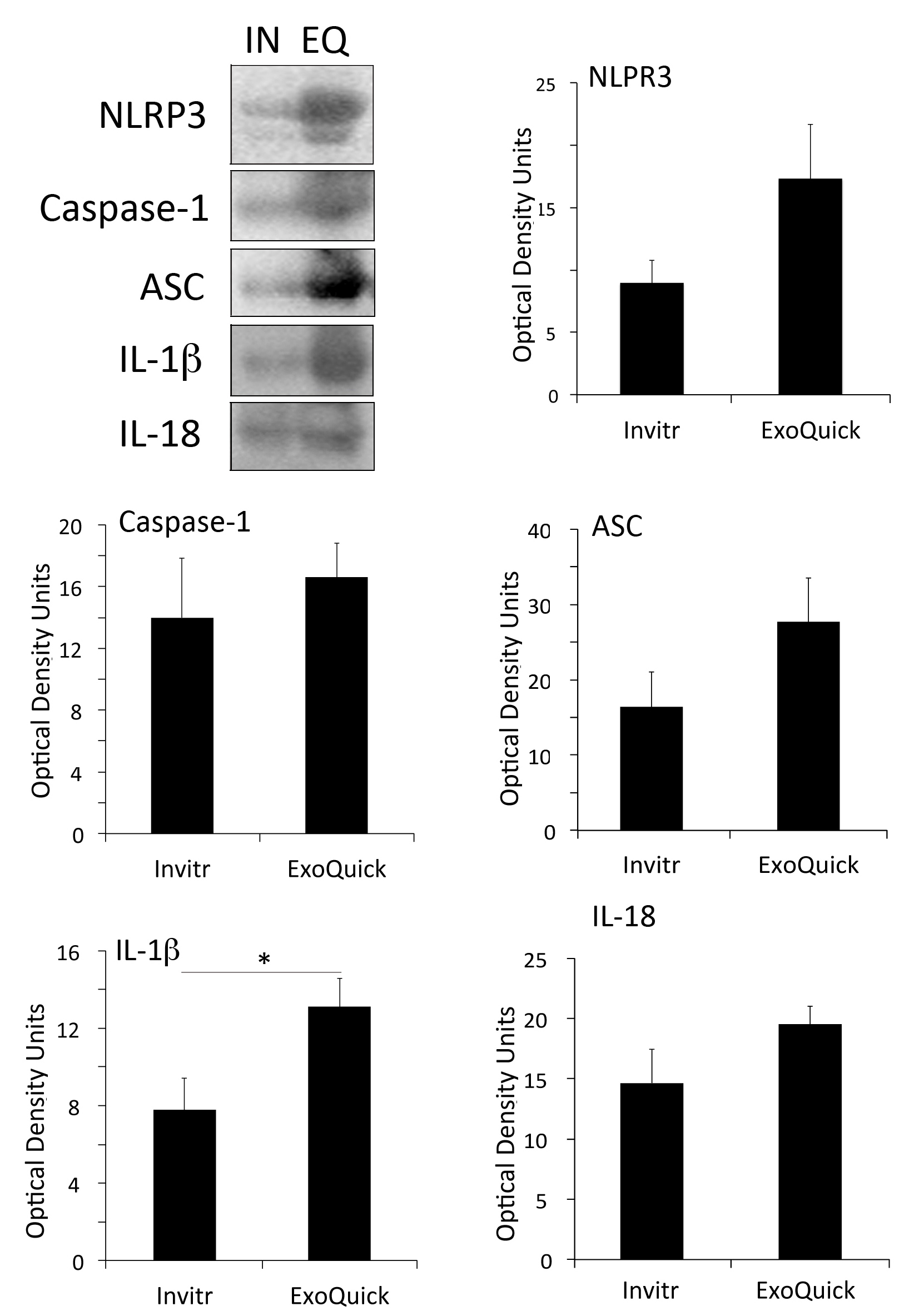

Supplement: Supplementary Figure 3 — Characterization of inflammasome proteins in serum-derived EV. Representative image of immunoblot analyses of inflammasome proteins in EV from serum. Quantification of immunoblot analysis of NLRP3, caspase-1, ASC, IL-1β, and IL-18 in EV derived from serum using the Invitrogen kit (IN) and the ExoQuick kit (EQ). Data presented as mean ± SEM. N = 6 per group. *p < 0.05. [file Image_3.JPEG]

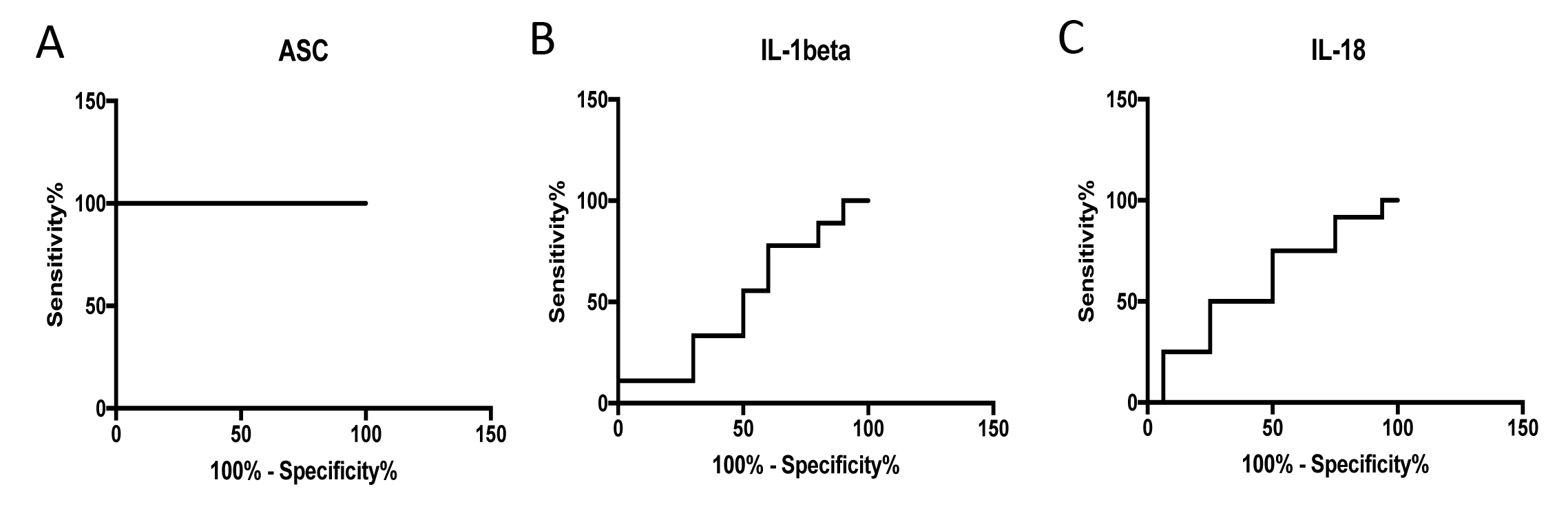

Supplement: Supplementary Figure 4 — ROC curves for ASC (A), IL-1β (B), and IL-18 (C) from serum samples of stroke and samples corresponding to the control subject group. [file Image_4.JPEG]
