# Supplementary material for: Inflammasome Proteins in Serum and Serum-Derived Extracellular Vesicles as Biomarkers of Stroke
Source: Front Mol Neurosci. 2018 Sep 4;11:309. doi: 10.3389/fnmol.2018.00309 (PMC6131639; doi:10.3389/fnmol.2018.00309)
Supplement: Supplementary Table 1 — Raw data used for the analysis of this paper. [file Table_1.DOCX]

**SERUM**

**ASC**

| Control | Stroke |
| --- | --- |
| 230.161 | 1734.735 |
| 249.906 | 656.636 |
| 283.705 | 1649.026 |
| 182.408 | 745.161 |
| 185.199 | 721.419 |
| 322.904 | 407.027 |
|  | 766.137 |
| 264.572 | 771.902 |
| 298.507 | 811.08 |
| 209.917 | 976.718 |
| 289.94 | 825.289 |
| 171.779 | 1551.523 |
| 303.014 | 1893.502 |
| 282.175 | 1160.935 |
| 151.356 | 1592.004 |
| 305.075 | 635.854 |
| 351.962 |  |
| 272.979 |  |
| 211.962 |  |
| 232.009 |  |
| 177.081 |  |
| 437.708 |  |
| 184.625 |  |
| 244.354 |  |
| 191.421 |  |
| 288.621 |  |
| 398.377 |  |
| 199.762 |  |
| 252.485 |  |
| 181.703 |  |
| 330.935 |  |
| 190.824 |  |
| 210.148 |  |
| 170.652 |  |
| 458.29 |  |
| 197.636 |  |
| 196.394 |  |
| 196.853 |  |
| 436.778 |  |
| 385.048 |  |
|  |  |
| 354.182 |  |
| 231.457 |  |
| 315.76 |  |
| 250.861 |  |
| 252.414 |  |
| 172.535 |  |
|  |  |
| 149.351 |  |
| 192.292 |  |
| 382.388 |  |
| 184.099 |  |
| 233.735 |  |
| 402.492 |  |
| 216.962 |  |
| 221.49 |  |
|  |  |
|  |  |
| 227.473 |  |
| 326.491 |  |
| 245.108 |  |
| 156.376 |  |
| 148.367 |  |
| 262.04 |  |
| 143.17 |  |
| 174.188 |  |
| 242.634 |  |
| 169.648 |  |
| 259.639 |  |
| 180.001 |  |
| 114.277 |  |
| 186.943 |  |
| 290.913 |  |
| 256.004 |  |
| 325.877 |  |
| 159.983 |  |
| 193.932 |  |
| 381.314 |  |
| 284.047 |  |
| 258.402 |  |

**Caspase-1**

| Control | Stroke |
| --- | --- |
| 1.399 | 6.426 |
| 1.08 | 3.101 |
| 1.277 | 3.005 |
| 1.869 | 1.011 |
| 3.464 | 1.12 |
| 1.522 | 2.028 |
|  | 1.963 |
| 2.703 | 1.424 |
| 1.102 | 7.798 |
|  | 7.662 |
|  | 7.316 |
|  | 8.178 |
|  | 2.639 |

**IL-18**

| Control | Stroke |
| --- | --- |
| 301.794 | 90.028 |
| 201.729 | 129.931 |
| 220.095 | 201.256 |
| 176.761 | 247.428 |
| 414.275 | 200.317 |
| 365.247 | 364.511 |
| 250.06 |  |
| 422.656 | 263.24 |
| 154.709 | 350.535 |
| 340.058 | 247.071 |
| 401.193 | 268.033 |
| 197.874 | 598.893 |
| 172.644 | 287.272 |
| 296.602 | 399.758 |
| 71.083 | 314.043 |
| 259.665 | 719.108 |
| 161.044 |  |
| 264.877 |  |
| 181.314 |  |
| 267.299 |  |
| 254.793 |  |
| 168.91 |  |
| 215.852 |  |
| 322.212 |  |
| 352.547 |  |
| 270.429 |  |
| 214.651 |  |
| 137.754 |  |
| 157.465 |  |
| 225.436 |  |
| 331.824 |  |
| 221.249 |  |
| 182.04 |  |
| 230.803 |  |
| 141.579 |  |
| 193.753 |  |
| 228.834 |  |
| 249.756 |  |
| 196.698 |  |
| 249.668 |  |
| 216.769 |  |
| 227.78 |  |
| 178.831 |  |
| 280.246 |  |
| 260.112 |  |
| 326.348 |  |
| 127.739 |  |
| 277.242 |  |
| 198.42 |  |
| 81.272 |  |
| 40.5 |  |
| 304.396 |  |
| 162.69 |  |
| 384.035 |  |
| 180.761 |  |
| 125.331 |  |
| 213.08 |  |
| 195.529 |  |
| 178.879 |  |
| 213.791 |  |
| 242.073 |  |
| 152.61 |  |
| 101.294 |  |
| 306.446 |  |
| 200.099 |  |
| 301.409 |  |
| 117.147 |  |
| 155.932 |  |
| 212.702 |  |
| 240.876 |  |
| 193.35 |  |
| 196.446 |  |
| 275.584 |  |
| 154.959 |  |
|  |  |
| 228.121 |  |
| 202.907 |  |
| 287.21 |  |
| 343.337 |  |
| 415.221 |  |

**IL-1beta**

| Control | Stroke |
| --- | --- |
| 0.522 | 2.138 |
| 3.276 | 1.208 |
| 1.021 | 0.754 |
| 0.648 | 0.413 |
|  | 0.627 |
|  | 0.514 |
| 0.875 | 0.947 |
| 1.747 | 1.907 |
|  |  |
| 1.897 |  |
| 0.617 |  |

**SERUM-DERIVED EXTRACELLULAR VESICLES**

**ASC**

| Control EV | Stroke EV |
| --- | --- |
| 47.869 | 519.155 |
| 38.801 | 139.226 |
| 43.962 | 463.577 |
| 52.624 | 138.845 |
| 31.64 | 172.609 |
| 49.158 | 107.885 |
| 44.053 | 138.537 |
| 87.252 | 147.977 |
| 45.554 | 427.397 |
| 30.988 | 232.67 |
| 75.943 | 121.914 |
| 26.127 | 467.934 |
| 63.573 | 455.162 |
| 44.708 | 295.489 |
| 36.122 | 290.33 |
| 49.547 | 158.495 |

**IL-18**

| Control EV | Stroke EV |
| --- | --- |
| 21.265 | 14.282 |
| 20.681 | 18.731 |
| 12.182 | 42.526 |
| 39.397 | 26.977 |
| 29.356 | 24.57 |
| 58.031 | 53.829 |
| 15.694 |  |
| 21.809 | 36.833 |
| 18.58 |  |
| 30.018 | 36.383 |
| 39.17 | 27.169 |
| 27.555 |  |
| 28.811 | 31.651 |
| 15.073 | 48.376 |
| 22.743 | 20.004 |
| 41.293 |  |

**IL-1beta**

| Control EV | Stroke EV |
| --- | --- |
| 0.658 | 0.673 |
| 0.467 | 1.194 |
| 0.553 | 0.758 |
| 0.431 |  |
| 0.413 | 0.6 |
| 0.603 | 0.423 |
| 0.485 | 0.564 |
| 0.952 | 0.545 |
| 1.139 | 0.455 |
| 0.886 | 0.516 |
